# High titers and low fucosylation of early human anti-SARS-CoV-2 IgG promote inflammation by alveolar macrophages

**DOI:** 10.1126/scitranslmed.abf8654

**Published:** 2021-05-11

**Authors:** Willianne Hoepel, Hung-Jen Chen, Chiara E. Geyer, Sona Allahverdiyeva, Xue D. Manz, Steven W. de Taeye, Jurjan Aman, Lynn Mes, Maurice Steenhuis, Guillermo R. Griffith, Peter I. Bonta, Philip J.M. Brouwer, Tom G. Caniels, Karlijn van der Straten, Korneliusz Golebski, René E. Jonkers, Mads D. Larsen, Federica Linty, Jan Nouta, Cindy P.A.A. van Roomen, Frank E.H.P. van Baarle, Cornelis M. van Drunen, Gertjan Wolbink, Alexander P.J. Vlaar, Godelieve J. de Bree, Rogier W. Sanders, Lisa Willemsen, Annette E. Neele, Diederik van de Beek, Theo Rispens, Manfred Wuhrer, Harm Jan Bogaard, Marit J. van Gils, Gestur Vidarsson, Menno de Winther, Jeroen den Dunnen

**Affiliations:** 1 Department of Rheumatology and Clinical Immunology, Amsterdam UMC, Amsterdam Rheumatology and Immunology Center, Meibergdreef 9, 1105 AZ Amsterdam, Netherlands.; 2 Department of Experimental Immunology, Amsterdam UMC, University of Amsterdam, Amsterdam Infection and Immunity Institute, Meibergdreef 9, 1105 AZ Amsterdam, Netherlands.; 3 Department of Medical Biochemistry, Experimental Vascular Biology, Amsterdam Cardiovascular Sciences, Amsterdam Infection and Immunity, Amsterdam UMC, University of Amsterdam, Meibergdreef 9, 1105 AZ Amsterdam, Netherlands.; 4 Department of Medical Microbiology, Amsterdam UMC, University of Amsterdam, Amsterdam Infection and Immunity Institute, Meibergdreef 9, 1105 AZ Amsterdam, Netherlands.; 5 Department of Pulmonary Medicine, Amsterdam UMC, location VUMC, De Boelelaan 1117, 1081 HV Amsterdam, Netherlands.; 6 Department of Experimental Immunohematology, Sanquin Research, Amsterdam, Netherlands, and Landsteiner Laboratory, Amsterdam UMC, University of Amsterdam, Plesmanlaan 125, 1066 CX Amsterdam, Netherlands.; 7 Department of Pulmonology, Amsterdam UMC, University of Amsterdam, Meibergdreef 9, 1105 AZ Amsterdam, Netherlands.; 8 Department of Internal Medicine, Amsterdam UMC, University of Amsterdam, Amsterdam Infection and Immunity Institute, Meibergdreef 9, 1105 AZ Amsterdam, Netherlands.; 9 Department of Respiratory Medicine, Amsterdam UMC, University of Amsterdam, Meibergdreef 9, 1105 AZ Amsterdam, Netherlands.; 10 Center for Proteomics and Metabolomics, Leiden University Medical Center, Albinusdreef 2, 2333 AZ Leiden, Netherlands.; 11 Department of Intensive Care Medicine, Amsterdam UMC, University of Amsterdam, Meibergdreef 9, 1105 AZ Amsterdam, Netherlands.; 12 Department of Otorhinolaryngology, Amsterdam UMC, University of Amsterdam, Meibergdreef 9, 1105 AZ Amsterdam, Netherlands.; 13 Department of Rheumatology, Amsterdam Rheumatology and immunology Center, Reade, Admiraal Helfrichstraat 1, 1056 AA Amsterdam, the Netherlands.; 14 Department of Immunopathology, Sanquin Research & Landsteiner Laboratory Academic Medical Centre, Plesmanlaan 125, 1066 CX Amsterdam, the Netherlands.; 15 Weill Medical College of Cornell University, 1300 York Avenue, New York, NY 10021, USA.; 16 Department of Neurology, Neuroscience, University of Amsterdam, Meibergdreef, Amsterdam UMC, Amsterdam, the Netherlands.

## Abstract

Patients diagnosed with coronavirus disease 2019 (COVID-19) become critically ill primarily around the time of activation of the adaptive immune response. Here, we provide evidence that antibodies play a role in the worsening of disease at the time of seroconversion. We show that early phase severe acute respiratory distress syndrome coronavirus 2 (SARS-CoV-2) spike protein-specific IgG in serum of critically ill COVID-19 patients induces excessive inflammatory responses by human alveolar macrophages. We identified that this excessive inflammatory response is dependent on two antibody features that are specific for patients with severe COVID-19. First, inflammation is driven by high titers of anti-spike IgG, a hallmark of severe disease. Second, we found that anti-spike IgG from patients with severe COVID-19 is intrinsically more pro-inflammatory because of different glycosylation, particularly low fucosylation, of the antibody Fc tail. Notably, low fucosylation of anti-spike IgG was normalized in a few weeks after initial infection with SARS-CoV-2, indicating that the increased antibody-dependent inflammation mainly occurs at the time of seroconversion. We identified Fcγ Receptor (FcγR) IIa and FcγRIII as the two primary IgG receptors that are responsible for the induction of key COVID-19-associated cytokines such as interleukin-6 and tumor necrosis factor. In addition, we show that anti-spike IgG-activated human macrophages can subsequently break pulmonary endothelial barrier integrity and induce microvascular thrombosis in vitro. Finally, we demonstrate that the inflammatory response induced by anti-spike IgG can be specifically counteracted by fostamatinib, an FDA- and EMA-approved therapeutic small molecule inhibitor of Syk kinase.

## Teaser

High titers and low fucosylation of human anti-spike protein IgG promote alveolar macrophage activation and inflammation.

## INTRODUCTION

Coronavirus disease 2019 (COVID-19), which is caused by severe acute respiratory syndrome coronavirus 2 (SARS-CoV-2), is characterized by mild flu-like symptoms in the majority of patients (*1*, *2*). However, approximately 20% of the cases have more severe disease outcomes, with bilateral pneumonia that may rapidly deteriorate into acute respiratory distress syndrome (ARDS) and even death by respiratory failure. With high numbers of infected people worldwide and limited treatments available, safe and effective therapies for the most severe cases of COVID-19 are urgently needed.

Remarkably, many of the COVID-19 patients with severe disease show a dramatic worsening of the disease around 1-2 weeks after onset of symptoms (*2*, *3*). This is suggested not to be a direct effect of viral infection, but instead to be caused by over-activation of the immune system in response to infection because worsening of disease coincides with the activation of adaptive immunity (*2*). This excessive immune response is frequently described as a ‘cytokine storm’, characterized by high concentrations of pro-inflammatory cytokines (*3*, *4*). A detailed assessment of the cytokine profile in severe cases of COVID-19 indicates that some cytokines and chemokines are strongly elevated, such as interleukin (IL)-6, IL-8, and tumor necrosis factor (TNF) (*5*–*7*). In contrast, type I and III interferon (IFN) responses, which are critical for early anti-viral immunity, appear to be suppressed (*8*, *9*). Altogether, the high pro-inflammatory cytokines, known to induce collateral damage to tissues, together with muted anti-viral responses suggest that an unfavorable immune response may be driving disease patients with severe cases of COVID-19.

Antibodies pose a potential candidate of the adaptive immune system that could explain the observed worsening of disease during SARS-CoV-2 infection. Previous studies on Dengue virus identified that IgG antibodies can increase the infection of cells by a process known as antibody-dependent enhancement (ADE) (*10*). However, thus far there is little evidence for antibody-enhanced infection in COVID-19 (*11*). In addition to ADE (which increases viral infection of cells), human IgG antibodies can also worsen pathology by increasing the release of pro-inflammatory cytokines. Initial studies identified this phenomenon in autoimmune disorders such as rheumatoid arthritis, where IgG auto-antibodies promote synovial inflammation (*12*, *13*). More recently, antibody-dependent inflammation has also been observed upon infection with SARS-CoV-1, and this was induced by anti-spike IgG (*14*). In both rheumatoid arthritis and SARS-CoV-1 infection, IgG antibodies convert wound healing “M2” macrophages to a pro-inflammatory phenotype (*12*, *14*, *15*). Combined, these data hint toward a pathogenic role for IgG in severe cases of COVID-19. In this study we explored the hypothesis that anti-SARS-CoV-2 antibodies drive excessive inflammation in severe cases of COVID-19 and define therapeutic approaches to suppress these responses.

## RESULTS

### High titers of anti-spike IgG promote inflammation by alveolar macrophages.

**Fig. 1:**
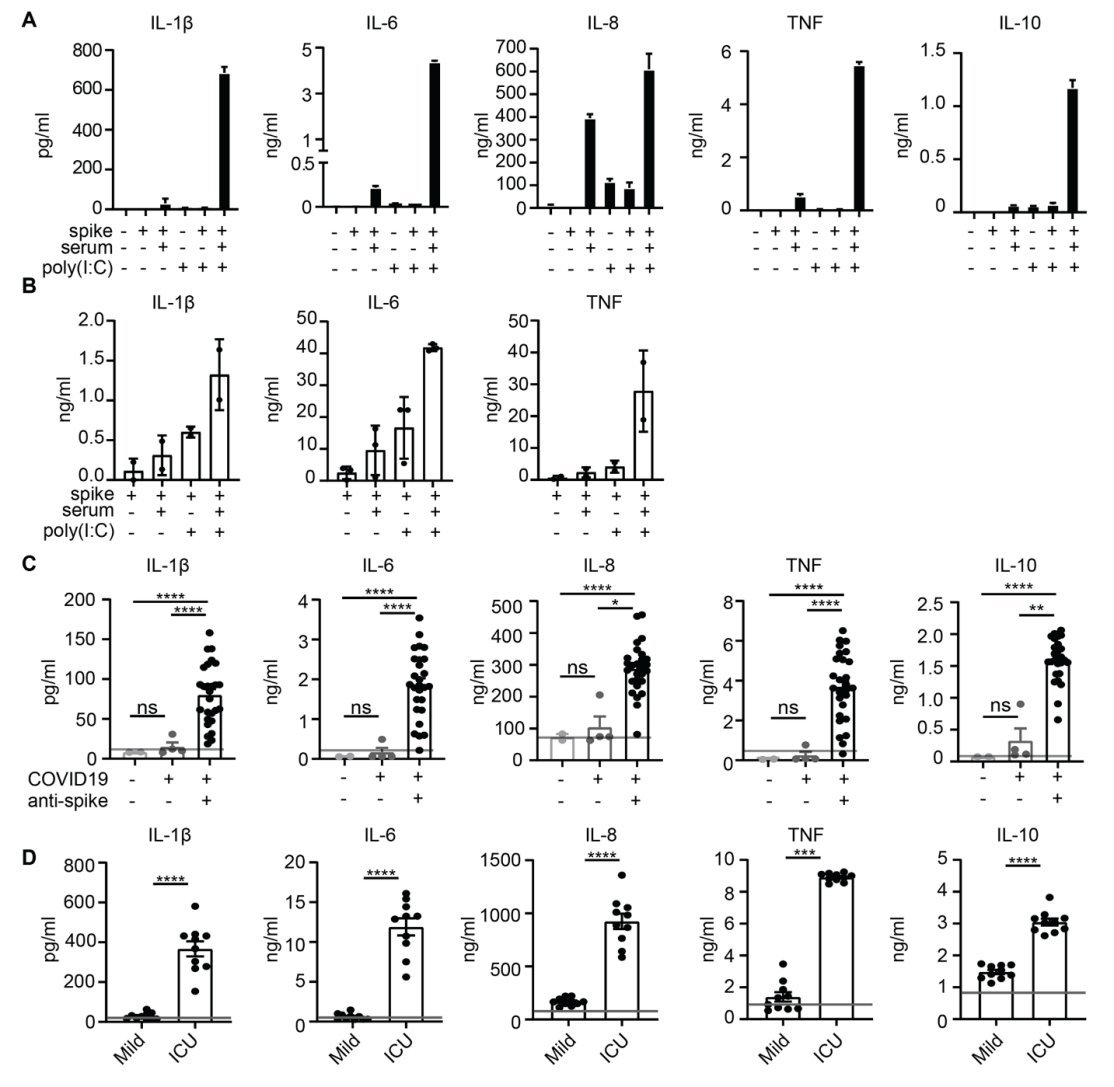
High titers of anti-spike IgG induce inflammation by macrophages. (A) Cytokine production by human macrophages after 24 hour stimulation with combinations of spike protein, COVID-19 serum (50x diluted), and poly(I:C). Triplicate values from a representative experiment with serum from five different COVID-19 patients and two different macrophage donors (mean+SD). (B) Cytokine production by primary alveolar macrophages obtained from BAL, stimulated as in (A). Every dot represents cytokine production after 24 hours by a different macrophage donor performed in triplicate (mean±SEM). (C) Macrophages stimulated with spike protein and poly(I:C) were co-stimulated with serum from patients in the ICU for lung disease that either did not have COVID-19 (n=2), had COVID-19 but were negative for anti-spike IgG (n=4), or had COVID-19 and were positive for anti-spike IgG (n=27). Horizontal grey line depicts cytokine induction upon stimulation with poly(I:C)+spike protein. Significance was calculated with Brown-Forsythe and Welch’s ANOVA test and corrected by Dunnett T3 test for multiple test correction (mean±SEM). (D) Macrophages stimulated with spike protein and poly(I:C) were co-stimulated with serum isolated from patients with mild COVID-19 (n=10) and were compared to serum from patients in the ICU for COVID-19 (n=10). Significant differences were calculated with an unpaired *t* test. Each dot represents cytokine production after 24 hours by macrophages stimulated with a different serum donor (mean±SEM). Horizontal grey line depicts cytokine induction upon stimulation with poly(I:C) plus spike protein. *P<0.05; **P<0.01; ***P<0.001; ****P<0.0001; ns=not significant.

We assessed the effect of anti-spike antibodies from serum of patients who were critically ill COVID-19 on human M2-polarized macrophages. Our previous transcriptional analysis revealed that macrophage colony-stimulating factor (M-CSF) plus interleukin (IL)-10 induces M2 monocyte differentiation that generates macrophages that most closely resemble primary human lung macrophages (*16*). Since activation of immune cells by IgG antibodies requires immune complex formation by binding of IgG to its cognate antigen (*17*, *18*), we generated spike-IgG immune complexes by incubating SARS-CoV-2 spike-coated wells with diluted serum from patients with severe COVID-19 treated in the intensive care unit at the Amsterdam University Medical Centers (UMC) that tested positive for anti-SARS-CoV-2 IgG (fig. S1A). Stimulation with spike protein alone did not induce cytokine production, whereas spike-IgG immune complexes elicited small amounts of IL-1β, IL-6, and tumor necrosis factor (TNF), but very high IL-8 production by human macrophages (Fig. 1A). However, since in the later phase of infection (1-2 weeks after initial exposure) lung macrophages are simultaneously exposed to virus-associated stimuli and anti-spike IgG immune complexes, we also assessed the effect of the combination of these two stimuli. Strikingly, combined stimulation of anti-spike IgG immune complexes and the toll-like receptor 3 agonist, polyinosinic:polycytidylic acid (poly(I:C)) increased the production of COVID-19-associated pro-inflammatory cytokines IL-1β, IL-6, and TNF compared to IgG or poly(I:C) alone (Fig. 1A). Similar effects were observed with other viral and bacterial co-stimuli (fig. S1B). Induction of the anti-inflammatory cytokine IL-10 was also increased (Fig. 1A), similar to what is observed in COVID-19 patients (*19*). We confirmed these findings in primary human alveolar macrophages that were obtained by bronchoalveolar lavage (BAL), which showed similar responses (Fig. 1B). Phenotypical analysis of these human alveolar macrophages showed significantly decreased expression of M2 markers upon co-stimulation with anti-spike IgG immune complexes, indicating the polarization toward a more inflammatory phenotype (*P* <0.0001 (*CD163), P* = 0.001 (*CD209*), fig. S1C).

To assess whether the inflammatory response is dependent on anti-spike antibodies, we compared the effect of sera from 33 intensive care lung disease patients that either (1) did not have COVID-19, (2) had COVID-19 but were still negative for anti-spike IgG, or (3) had COVID-19 and were positive for anti-spike IgG (table S1). Whereas serum of non-COVID-19 patients and anti-spike IgG-negative COVID-19 patients showed no up-regulation of pro-inflammatory cytokines compared to individual poly(I:C) stimulation, IL-1β, IL-6, IL-8, and TNF production was amplified by serum of COVID-19 patients with anti-spike IgG (*P* <0.0001, Fig. 1C). To further confirm that the observed inflammation is induced by anti-spike IgG, and not by other inflammatory components in serum, we purified IgG from serum of critically ill COVID-19 patients that were seropositive and healthy controls that were seronegative for anti-SARS-CoV-2. Whereas pro-inflammatory cytokine production was strongly amplified by purified IgG from severely ill COVID-19 patients, no amplification was observed by purified IgG from controls (fig. S1D).

To determine whether the inflammatory responses are specific for severely ill COVID-19 patients, or are also induced by patients that have mild symptoms, we directly compared cytokine amplification by serum obtained from patients with mid COVID-19 or patients in the ICU (table S2). Amplification of pro-inflammatory cytokine production was specific for severely ill patients (*P* <0.0001, Fig. 1D), which was in line with the substantially lower anti-spike titers in mild patients (fig. S1E), whereas the fucosylation was comparable (fig. S1F).

**Fig. 2:**
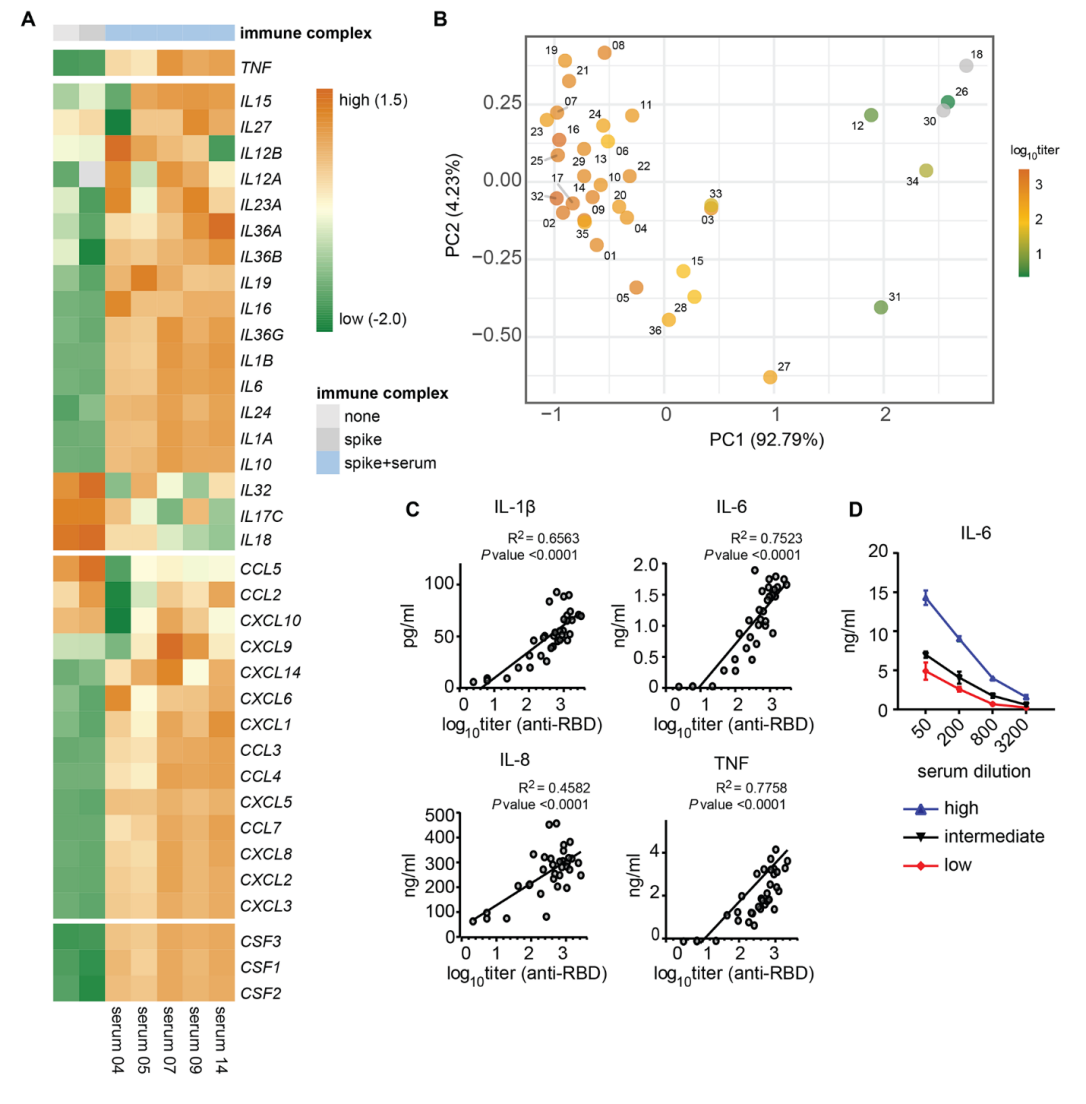
High titers of anti-spike IgG induce inflammation by macrophages. (A) Heatmap showing scaled log2 expression (z-score) of genes assessed by RNA sequencing after a 6 hour stimulation of human macrophages with poly(I:C) with or without spike protein and serum from five patients with COVID-19 that tested positive for anti-spike IgG. (B) Principal component analysis of the combined cytokine profile (IL-1β, IL-6, IL-8, IL-10, TNF, IFN-β, IFN-γ, CXCL10) for all serum samples overlaid with log10 anti-RBD IgG titers. Titers of each serum sample is represented by the color scale. Samples with anti-RBD IgG titer below detection limit were colored gray. Numbers represent the patients sample number. (C) Correlation graphs of anti-RBD IgG titer from COVID-19 serum against cytokine production of macrophages after stimulation. The square of Pearson correlation coefficient (R^2^) and P-value are stated in each graph. (D) Macrophages stimulated with spike protein and poly(I:C) were co-stimulated with different dilutions of serum from patients with varying anti-spike titers. Titers were from patient 2 (high titer), 5 (intermediate titer) or 6 (low titer). IL-6 production was determined after 24 hours.

RNA sequencing analysis of macrophages stimulated with sera from anti-spike IgG positive COVID-19 patients showed induction of a pro-inflammatory gene program, as highlighted by induction of *TNF*, interleukins, chemokines, and macrophage differentiation factors (Fig. 2A). Interestingly, Interferon (IFN)-β and IFN-γ were induced also by anti-spike positive serum, whereas the classical downstream interferon response gene, CXCL10, was reduced (*P* <0.0001, fig. S1G to I), which is in line with recent findings by others (*20*).

In patients with COVID-19, high anti-spike IgG titers are strongly associated with disease severity (*21*, *22*). To determine whether anti-spike titers correlate with higher cytokine responses by human macrophages, we performed a principal component analysis (PCA) of the combined cytokine production data for all samples that, upon overlaying with anti-spike IgG titers, suggested that the inflammatory response of macrophages was associated with IgG titers (Fig. 2B). Subsequent analysis similarly demonstrated that anti-receptor binding domain (RBD) IgG titers and cytokine production correlate for the cytokines IL-1β (*P* <0.0001), IL-6 (*P* <0.0001), IL-8 (*P* <0.0001), IL-10 (*P* <0.0001), and TNF (*P* <0.0001, Fig. 2C and fig. S1J). Similar correlations were observed for IL-6 and total anti-spike IgG (*P* <0.0001, fig. S1K). IFN-β (*P* = 0.0004) and IFN-γ (*P* <0.0001) also showed a positive correlation, whereas CXCL10 showed a negative correlation (*P* <0.0001, fig. S1J), which may be related to reduced expression of IFN receptors (fig. S1L). Stimulation with immune complexes made from three serum samples with different titers using serial-step dilutions showed a dose-dependent induction of pro-inflammatory cytokines (Fig. 2D), thereby confirming that high anti-spike titers drive pro-inflammatory cytokine production by human macrophages.

To assess whether inflammatory responses are induced directly upon virus opsonization, or whether this requires spike expression by infected cells, we stimulated macrophages with anti-spike IgG-opsonized pseudo-typed virus. Virus opsonization had no detectable effect on cytokine production (fig. S1M), which is in line with previous findings that small IgG immune complexes are unable to trigger cytokine production (*23*). In contrast, IgG-opsonized spike-expressing 293F cells, which mimic SARS-CoV-2 infected cells and induce the formation of larger immune complexes, did amplify IL-6 production by macrophages (fig. S1N). These results indicate that anti-spike induced inflammation requires large IgG immune complexes, as occurs upon host cell infection. Combined, these data demonstrate that high titers of anti-spike IgG from serum of severely ill COVID-19 patients induce a strong pro-inflammatory response by otherwise immunosuppressive human M2 macrophages, which is characterized by production of classical cytokine storm mediators such as IL-1β, IL-6, IL-8, and TNF.

### Aberrant glycosylation of anti-spike IgG contributes to inflammation.

**Fig. 3:**
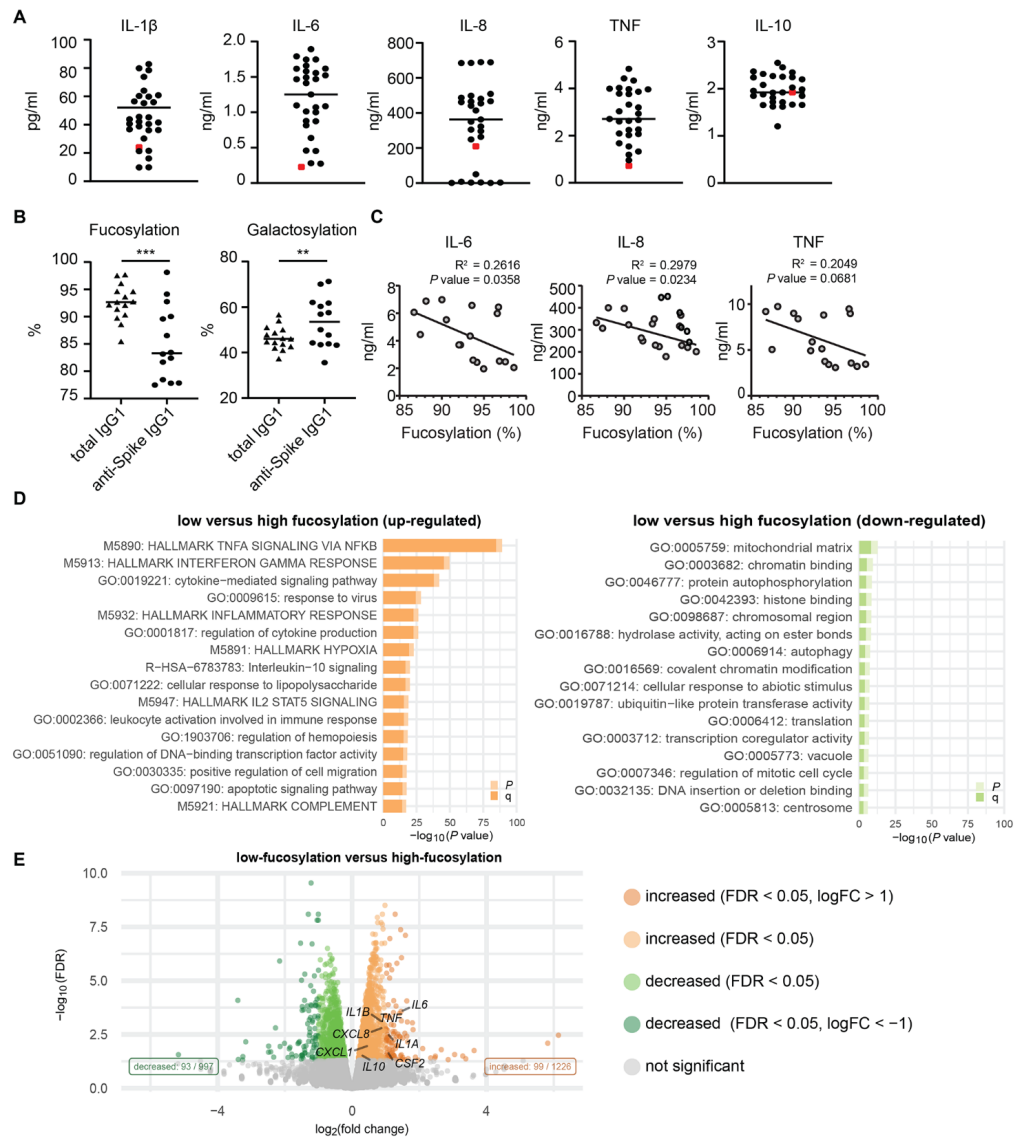
Low fucosylation of IgG correlates with enhanced anti-spike IgG-induced inflammation. (A) Macrophages stimulated with spike protein and poly(I:C) were co-stimulated with either 50x diluted serum from different anti-spike^+^ patients with COVID-19 (black dots), or with recombinant anti-spike antibody COVA1-18 (red dot). Representative example of 4 independent macrophages donors is shown. Cytokine production was measured after 24 hours. (B) Fucosylation and galactosylation of total and anti-spike specific IgG1 antibodies. Statistics were calculated with a paired *t* test. **P < 0.01; ***P <0.001. (C) Correlation graphs of fucosylation percentages of anti-spike IgG1 from COVID-19 serum against cytokine production of macrophages after stimulation. The square of Pearson correlation coefficient (R^2^) and *P*-value are stated in each graph. (D) Pathway analysis of differentially expressed genes (DEGs). DEGs were defined by a false discovery rate (FDR) <0.05 and an absolute log_2_ fold-change higher or lower than 0. Pathway enrichment analyses were performed using the Metascape on 2020-07-29. p=*P*-value; q=FDR-corrected *P*-value. (E) Volcano plot depicting up- and down-regulated genes when comparing macrophages stimulated for 6 hours with spike, poly(I:C), and serum with low-fucosylated IgG to the same stimulation with high-fucosylated IgG.

In addition to the anti-spike antibodies from serum, we tested the effect of the recombinant anti-spike IgG COVA1-18, which we generated previously from B cells isolated from a patient with COVID-19 (*24*). We stimulated macrophages with anti-spike immune complexes made with a high concentration of recombinant anti-spike antibody COVA1-18 (mimicking a serum concentration of 100 μg/mL in our assay). This concentration is higher than the average anti-SARS-CoV-2 IgG concentration in patients with severe COVID-19, which according to previous studies on average peaks at 16.5 μg/mL (*25*). The high concentration of COVA1-18 immune complexes elicited substantially less IL-1β, IL-6, and TNF than anti-spike immune complexes made from COVID-19 serum (Fig. 3A). Interestingly, we did not observe this difference for the induction of anti-inflammatory cytokine IL-10 (Fig. 3A). These data suggest that the anti-spike IgG in severe cases of COVID-19 patients is intrinsically more pro-inflammatory than a recombinant IgG against the same target.

One of the critical characteristics that determines IgG pathogenicity is the glycosylation of the IgG Fc tail at position 297 (*26*, *27*). Recently, we and others have shown that anti-spike IgG of patients with severe COVID-19 have aberrant fucosylation and galactosylation, both compared to the total IgG within these individual patients, as well as compared to anti-spike IgG from mild or asymptomatic patients (*28*, *29*). We determined the glycosylation pattern of a subset of COVID-19 serum samples in the present study, which showed significantly decreased fucosylation (*P* = 0.0003) and increased galactosylation of anti-spike IgG compared to total IgG within the tested patients (*P* = 0.0096, Fig. 3B), similar to the study of Larsen *et al*. (*28*). Notably, fucosylation of anti-spike IgG correlated negatively with macrophage production of pro-inflammatory cytokines IL-6 (*P* = 0.0358) and IL-8 (*P* = 0.0234, Fig. 3C). No correlation was observed for TNF, IL-1β, or IL-10 (Fig. 3C). CXCL10 showed a positive correlation (*P* = 0.0443, fig. S2A). RNA sequencing data from patients with relatively low fucosylation (sera 07, 09, 14) and with patients with relatively normal fucosylation (sera 04 and 05) showed a very pronounced induction of inflammatory mediators and pro-inflammatory pathways specifically in low fucosylation patients (Fig. 3D and 3E).

**Fig. 4:**
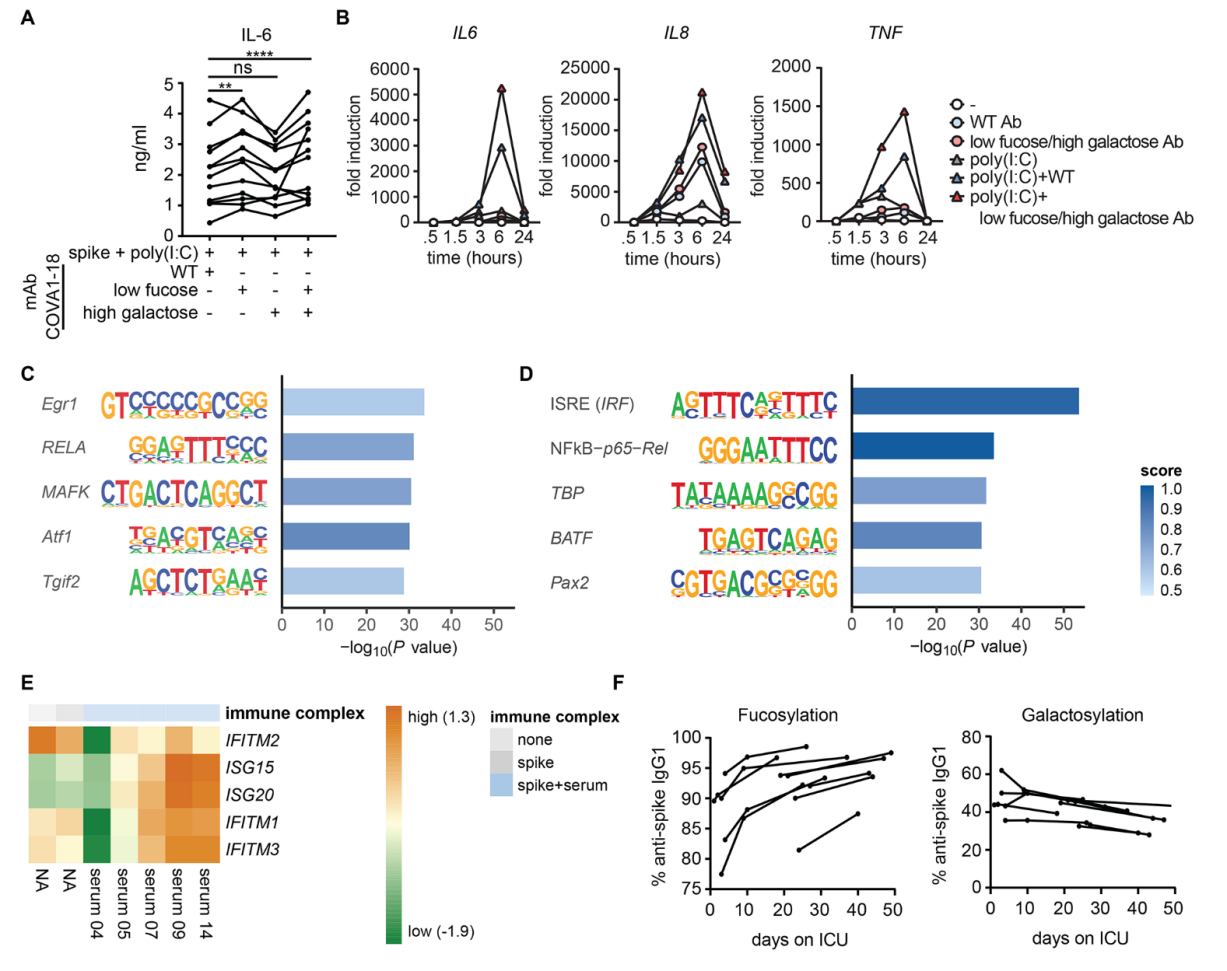
Low fucosylation of IgG promotes inflammatory cytokine production. (A) Macrophages stimulated with spike protein were co-stimulated with combinations of poly(I:C), COVA1-18 (wild-type, recombinant anti-spike IgG1), or COVA1-18 that had been modified to express low fucose or high galactose. IL-6 production was measured after 24 hours. Each line represent 1 macrophage donor, preformed in triplicate. Statistics were calculated with two way ANOVA. **P < 0.01; ****P <0.0001; ns, not significant. (B) Time-dependent fold changes (to 0 hour unstimulated M2 macrophages) in gene expression were depicted in line chart for *IL6*, *IL8*, and *TNF*. Macrophages stimulated with poly(I:C) were co-stimulated with immune complexes of wild-type antibody or an antibody that had been modified to express low fucose or high galactose. Representative example of 6 independent macrophages donors is shown. Cytokine production was measured after 0.5, 1.5, 3, 6 and 24 hours.(C) Enriched motifs for significantly up-regulated genes when comparing macrophages stimulated for 6 hours with spike and poly(I:C), with or without anti-spike IgG. (D) Enriched motifs for significantly up-regulated genes when comparing macrophages stimulated for 6 hours with spike, poly(I:C), and serum with low-fucosylated IgG to the same stimulation with high-fucosylated IgG. (E) Heatmap showing scaled log2 expression (z-score) of IFN-stimulated genes assessed by RNA sequencing after a 6 hour stimulation of human macrophages with poly(I:C) with or without spike protein and serum from 5 sero-positive patients with COVID-19. (F) IgG1 fucosylation and galactosylation of total and anti-spike specific antibodies was determined in serum samples over time for patients in the ICU with COVID-19. Each line represents one donor.

To determine whether anti-spike glycosylation directly modulates cytokine induction we stimulated macrophages with regular monoclonal COVA1-18, or modified COVA1-18 that had low fucosylation or high galactosylation (table S3). COVA1-18 with low fucosylation showed an increased capacity for amplification of pro-inflammatory cytokines (*P* <0.0001, Fig. 4A). High galactosylation alone or in combination with low IgG fucosylation did not lead to elevated cytokine production (Fig. 4A). COVA1-18 with low fucose and high galactose showed a similar amplification of *IL6*, *IL8*, and *TNF* mRNA expression over time (Fig. 4B), whereas *CXCL10* mRNA expression was again inhibited (fig. S2B). To gain more insight into the molecular mechanisms that underlie the enhanced inflammatory response induced by anti-spike IgG with aberrant glycosylation, we focused on the transcriptional responses induced by the anti-spike IgGs. Motif analyses of genes differentially induced by anti-SARS-CoV-2 monoclonal IgG COVA1-18 showed clear enrichment for classical inflammatory transcription factors like EGR, p65 (RELA) and Maf (Fig. 4C). Interestingly, upon comparison of genes affected by the differential glycosylation in patient samples, we identified Interferon Stimulated Responses Elements (ISREs) as a key enriched motif (Fig. 4D), suggesting amplification of macrophage activation via interferon pathways. This was further indicated by increased IFN-β and IFN-γ secretion (fig. S2C) by afucosylated IgG compared to IgG with normal fucosylation, and by increased expression of a series of classical interferon response genes (*30*) (Fig. 4E). These data suggest that afucosylated anti-SARS-CoV-2 IgG promotes inflammation by engagement of IFN pathways, which are classical co-factors to promote macrophage activation (*31*).

Finally, we determined whether the aberrant glycosylation pattern of anti-spike IgG is stable over time, by analyzing fucosylation and galactosylation over time of the patients in our study. Both fucosylation and galactosylation normalized within days to weeks after ICU admission (Fig. 4F). Similar results were observed for the other types of IgG glycosylation (fig. S2D). These data indicate that, in patients critically ill with COVID-19, the first anti-spike IgG antibodies that are produced after infection are intrinsically more inflammatory by bearing different glycosylation patterns.

### Anti-spike IgG induces activation of endothelium and platelets in vitro.

**Fig. 5:**
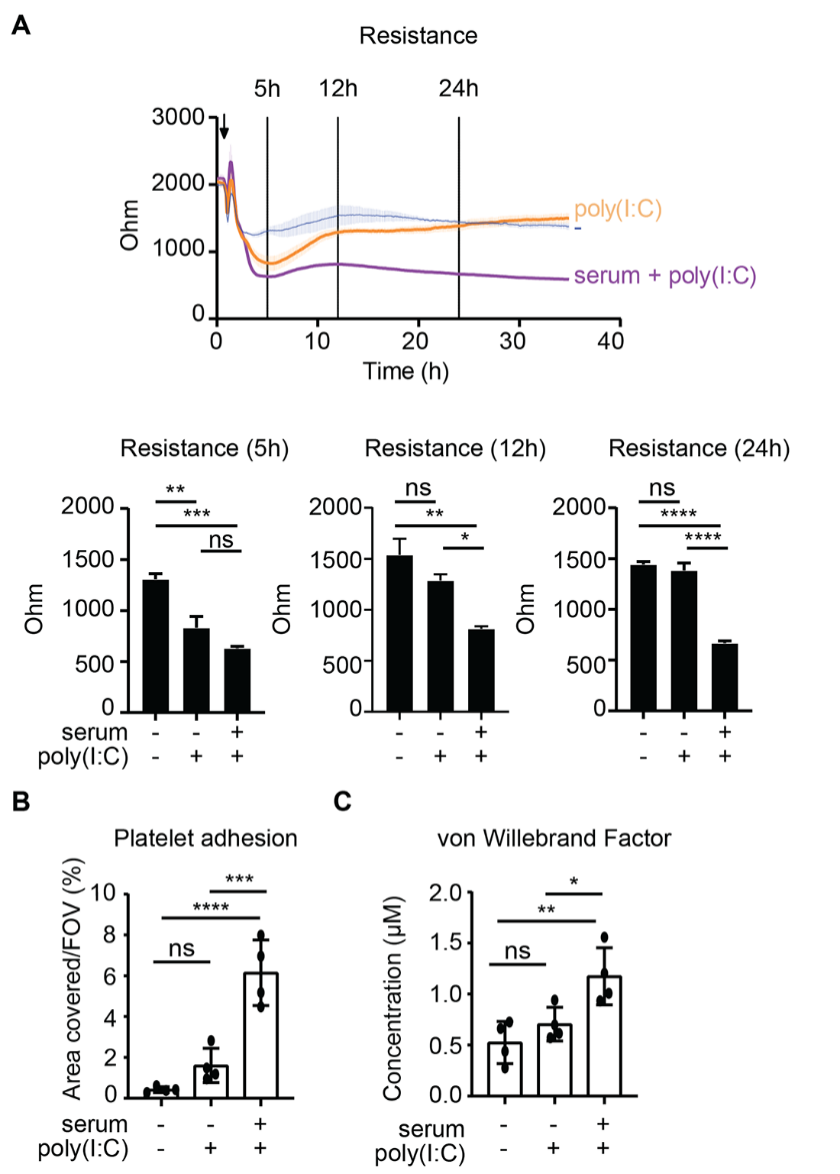
Anti-spike IgG breaks endothelial barrier integrity and activates platelets in vitro. (A) Human pulmonary arterial endothelial cells were exposed to supernatants of macrophages that were unstimulated or had been stimulated with poly(I:C) and spike protein, with or without serum from patients with COVID-19. Endothelial barrier integrity was quantified by measuring the resistance over time using electrical cell-substrate impedance sensing. Statistics were calculated using an ordinary one-way ANOVA and corrected with Tukey’s comparisons test. (B) Endothelium stimulated as in (A) for 24 hours was perfused with platelets for 5 min, after which the area covered by platelets was quantified. FOV, field of view. (C) Flow supernatant was collected after perfusion under B, and von Willebrand Factor concentrations were measured with ELISA. Statistics were calculated using an ordinary one-way ANOVA and corrected using Sidak’s multiple comparison test. *P<0.05; **P<0.01; ***P<0.001; ****P<0.0001; ns=not significant.

The excessive lung inflammation in severely ill COVID-19 patients often leads to pulmonary edema, following disruption of the microvascular endothelium (*32*), and coagulopathy, which in many patients is characterized by pulmonary thrombosis (*33*). To test whether the excessive macrophage activation by anti-spike IgG may contribute to pulmonary edema and thrombosis, we applied in vitro models for endothelial barrier integrity (*34*) and in situ thrombosis (*35*) using primary human pulmonary artery endothelial cells (HPAEC), where thrombocytes are added under flow conditions. For this, we stimulated macrophages and employed the supernatant to assess endothelium and platelet activation. Although conditioned medium of poly(I:C)-stimulated macrophages induced only a transient drop in endothelial barrier integrity, co-stimulation of macrophages with spike protein and serum isolated from patients with severe COVID-19 induced long-lasting endothelial barrier disruption (Fig. 5A). In addition, during platelet perfusion we observed significantly increased platelet adhesion to endothelium exposed to conditioned medium of macrophages that had been co-stimulated with spike protein and serum (*P* <0.0001, Fig. 5B). This effect was paralleled by an increase in von Willebrand Factor release from the endothelial cells (Fig. 5C), indicative of an active pro-coagulant state of the endothelium. These data suggest that anti-spike IgG-induced inflammation by macrophages may contribute to permeabilization of pulmonary endothelium, microvascular thrombosis, and subsequent severe pulmonary problems.

### Fostamatinib counteracts inflammation induced by anti-spike IgG.

**Fig. 6:**
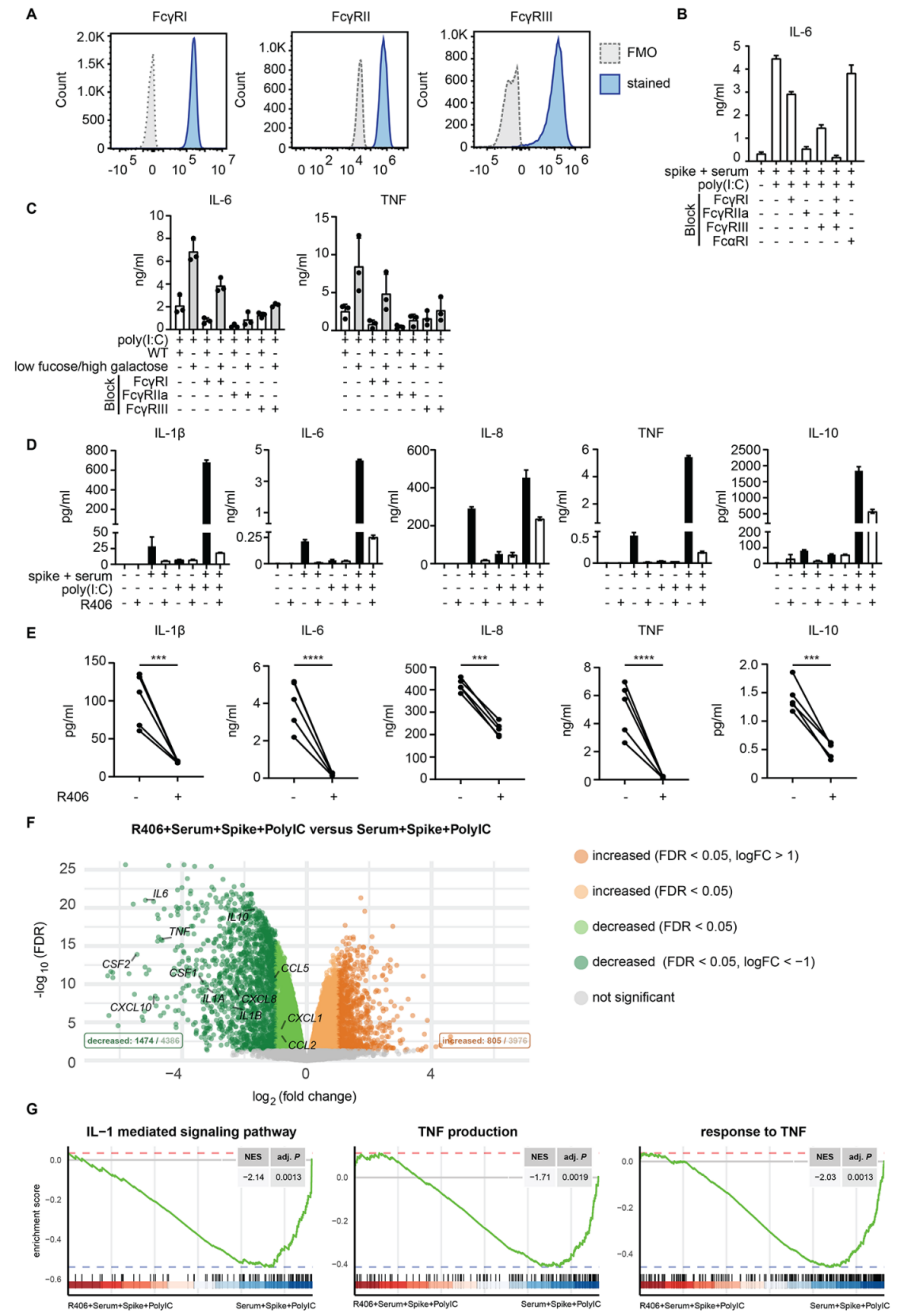
Anti-spike IgG-induced inflammation is FcγR dependent and can be counteracted by fostamatinib. (A) Membrane expression of FcγRI, FcγRII, and FcγRIII by human macrophages was determined by flow cytometry. FMO, fluorescence minus one control. (B) FcγRI, FcγRII, FcγRIII, or FcαRI were blocked by specific antibodies, after which macrophages were stimulated with spike, COVID-19 serum, poly(I:C), or a combination. IL-6 production was measured after 24 hours. Triplicate values from a representative experiment with serum from 3 different patients with COVID-19 and two different macrophage donors (mean±SD). (C) FcγRI, FcγRII, and FcγRIII were blocked by specific antibodies, after which macrophages stimulated with poly(I:C) and immune complexes of wild-type antibody or an antibody that had been modified to express low fucose and high galactose. Each dot represents cytokine production after 24 hours by a different macrophage donor (mean±SEM). (D and E). Macrophages were pre-incubated with Syk inhibitor R406, after which cells were stimulated as in (B). Cytokine production was measured after 24h. A representative donor is shown (D) and data are presented as mean±SD. The response for multiple donors with or without pre-incubation with R406 is shown (E). Every pair of dots represents cytokine production after 24 hours by a different serum donor. Statistics were calculated with a ratio paired *t* test. ***P<0.001; ****P<0.0001. (F) Volcano plot depicting up- and down-regulated genes when comparing macrophages stimulated for 6 hours with spike, poly(I:C), and serum to the same stimulation in the presence of R406. FDR=false discovery rate. (G) Gene set enrichment analysis (GSEA) of curated gene sets suppressed by R406: interleukin-1-mediated signaling pathway (GO:0070498), TNF production (GO:0032640), response to TNF (GO:0034612). NES stands for normalized enrichment score and adj. *P* represents the Benjamini-Hochberg (BH)-adjusted *P*-value.

Anti-spike IgG from severely ill COVID-19 patients promoted inflammatory cytokines, endothelial barrier disruption, and microvascular thrombosis in vitro, which are key phenomena underlying pathology in patients with severe COVID-19. Hence, counteracting this antibody-induced aberrant immune response could be of potential therapeutic interest. To determine how to counteract this antibody-dependent inflammation, we first set out to investigate which receptors on human macrophages are activated by the anti-SARS-CoV-2 IgG immune complexes. IgG immune complexes can be recognized by Fc gamma receptors (FcγRs), which includes FcγRI, FcγRIIa, and FcγRIII (*18*), which are all expressed on our human M2 macrophages (Fig. 6A). To determine whether FcγRs are involved in activation by anti-spike immune complexes, we blocked the different FcγRs with specific antibodies during stimulation, and analyzed cytokine production following exposure to anti-spike immune complexes. All FcγRs contributed to anti-spike-induced cytokine induction, but the most pronounced inhibition was observed upon blockade of FcγRIIa (Fig. 6B). No inhibition was observed upon blocking of Fc alpha receptor I (FcαRI), suggesting that IgA does not play a substantial role in the observed cytokine induction (Fig. 6B).

Since changes in glycosylation of the Fc tail can differentially affect the interaction of IgG with the different FcγRs, we also blocked the three different FcγRs upon co-stimulation with monoclonal IgG that either had conventional Fc glycosylation or low fucose and high galactose. Cytokine induction by both IgG with conventional glycosylation and low fucose and high galactose were mostly dependent on FcγRIIa (Fig. 6C). However, FcγRIII appeared to be the primary receptor responsible for the enhanced cytokine production by aberrant IgG glycosylation, since blocking FcγRIII specifically counteracted IL-6 and TNF production induced by IgGs with low fucose and high galactose (Fig. 6C). Interestingly, we did not observe this for IL-1β (fig. S3A), which may be related to activation of caspase-1 that is known to be mainly dependent on FcγRIIa (*36*).

FcγRs are known to induce signaling that critically depends on the kinase Syk (*12*, *36*). To determine whether we could counteract anti-spike-induced immune activation, we blocked Syk using R406, the active component of the small molecule inhibitor fostamatinib, an FDA- and EMA-approved drug for treatment of immune thrombocytopenia (ITP) (*37*). Strikingly, R406 significantly reduced pro-inflammatory cytokine production induced by anti-spike IgG from patients with severe COVID-19 (*P* <0.0001, Fig. 6D and E). Notably, inhibition by R406 appeared to be specific, since it selectively blocked anti-spike induced amplification of cytokines, but did not substantially affect cytokine production induced by poly(I:C) alone (Fig. 6D). Similar effects were observed with primary human macrophages obtained from BAL fluid (fig. S3B).

To assess the consequences of inhibition by fostamatinib in greater detail, we analyzed the effects of R406 on macrophages stimulated with spike, serum from COVID-19 patients, and poly(I:C) by RNA sequencing. In total 4386 genes were suppressed by R406 treatment, whereas 3976 genes were induced (false discovery rate < 0.05, Fig. 6F). Many of the classical pro-inflammatory mediators were present in the list of genes down-regulated by R406 treatment, including *TNF*, *IL1B*, *IL6,* and *CCL2*. Pathway analyses showed no clear pathways in the up-regulated genes, although suppressed genes were linked to inflammatory pathways (fig. S3C). Finally, gene set enrichment analysis (GSEA) showed that genes associated with several pro-inflammatory pathways, including IL-1 signaling and TNF production and response, were significantly down-regulated by R406 (*P* = 0.013, Fig. 6G). Response to type I IFN, Fc-gamma receptor signaling, glycolysis, and platelet activation gene sets were suppressed (fig. S3D). These data demonstrate that the excessive inflammatory response by anti-spike IgG from severely ill COVID-19 patients can be counteracted by the Syk inhibitor fostamatinib.

## DISCUSSION

It is still not well understood why many COVID-19 patients become critically ill around the time of activation of adaptive immune responses. Here, we identified the induction of pathogenic IgG antibody responses against the spike protein as a potential cause, which amplifies pro-inflammatory responses by human macrophages, and also induces subsequent endothelial barrier disruption and thrombosis (Fig. S4). The induction of inflammation by anti-SARS-CoV-2 IgG is both dependent on anti-spike IgG titers and on low fucosylation of these antibodies, which increases their inflammatory potential, most likely by over-activation through FcγRIII. During the course of infection, both these inflammatory parameters change. Anti-spike IgG titers rapidly increase after seroconversion followed by a gradual decline (*38*). In contrast, only the first wave of anti-spike IgG displays aberrant Fc glycosylation (characterized by low fucose and high galactose), which rapidly normalizes in the following days to weeks. Based on these two parameters, the induction of excessive inflammation by anti-spike IgG is particularly likely to occur in the days right after seroconversion, when titers are high and glycosylation is most aberrant. This correlates with the observed pathology in severely ill COVID-19 patients, which show a peak in inflammation, edema, and thrombosis around the time of seroconversion (*2*, *3*). In addition, this also correlates with the common absence of excessive inflammation in people that become re-infected with SARS-CoV-2 (*39*, *40*), since the anti-spike IgG in these re-infected individuals will have lower titers and most likely will have normalized Fc glycosylation.

In general, antibodies are beneficial for host defense by providing various mechanisms to counteract infections, including pathogen neutralization, phagocytosis, complement activation, antibody-dependent cellular cytotoxicity (ADCC), and cytokine production (*41*). These different effector functions of antibodies are induced to a greater or lesser extent depending on antibody-intrinsic characteristics, such as isotype, subclass, allotype, and glycosylation (*26*). In patients who are severely ill with COVID-19, the glycosylation of anti-spike IgG is changed, which can lead to pathology by over-activation of IgG effector functions, as we show here by particularly amplifying the production of COVID-19-associated cytokines such as IL-6 and TNF (*5*, *42*). Decreased IgG fucosylation, as observed in severe cases of COVID-19, has previously been observed in patients infected with human immunodeficiency virus (HIV) or Dengue virus (*43*, *44*), and may actually be a general phenomenon in a response to enveloped viruses (*28*). For Dengue virus, decreased IgG fucosylation has been described to contribute to worsening of the course of the disease after re-infection (*10*). Yet, it is important to realize that the underlying mechanism by which low-fucose IgG contributes to disease exacerbation is very different between Dengue virus and SARS-CoV-2. In Dengue virus infections, decreased IgG fucosylation worsens the pathology by binding to the virus and increasing the infection of host cells through enhanced uptake by FcγRs, a process known as antibody-dependent enhancement (ADE) (*10*). For SARS-CoV-2, there is very little evidence for ADE. Instead, increased pathology by afucosylated IgG in COVID-19 patients likely results from excessive immune activation. To make this difference clear, we propose to not use the term ADE, but instead to use antibody-dependent inflammation (ADI) to denote antibody-induced pathology as observed in COVID-19 patients.

The combination of decreased fucosylation and increased galactosylation of IgG is known to increase the affinity for FcγRIII (*26*). Whereas FcγRIII was indeed the primary receptor responsible for the inflammatory responses that were specifically induced by IgG with low fucose and high galactose, FcγRIIa contributed most to anti-spike-induced inflammation overall. These findings indicate that collaboration between multiple FcγRs is required for ADI by anti-spike IgG. The observed FcγR-dependent over-activation of human alveolar macrophages, which generally have a wound-healing, M2 phenotype, is in line with the general concept that the effect of ADI is most pronounced in immune cells that have a tolerogenic or anti-inflammatory phenotype, such as synovial M2 macrophages (*12*) or intestinal CD103^+^ dendritic cells (*45*). Although we focused on alveolar macrophages in this study, FcγRII and FcγRIII are also expressed by various other myeloid immune cells that are found in the inflamed lungs of patients with severe COVID-19, such as monocytes and neutrophils (*3*, *46*). Interestingly, over-activation of neutrophils by COVID-19 patient plasma can also be inhibited by fostamatinib (*47*). In addition, the high degree of aberrantly glycosylated anti-spike IgG could also contribute to pathology by activating non-immune cells. For example, airway epithelial cells express FcγRIII, are one of the main target cells of infection by SARS-CoV-2, closely interact with activated macrophages (*48*), and are a major source of IL-6 (*49*). In addition, anti-spike IgG may activate platelets through FcγRIIa (*50*, *51*), which would provide a direct way of platelet activation in addition to the indirect activation by macrophages and pulmonary endothelium that we observed in this study, thereby further promoting microvascular thrombosis.

It is still unclear how severe SARS-CoV-2 infections lead to the generation of IgG antibodies with aberrant glycosylation. Regarding the total amount of IgG in circulation, changes in glycosylation are associated with age and sex, which results in slightly decreased IgG fucosylation with age (*52*, *53*). However, in severely ill patients with COVID-19, it is specifically the anti-spike IgG that shows lower fucosylation. Although production of afucosylated IgG seems to be a general mechanism in response to enveloped viruses (*28*), it is unclear why afucosylation is more pronounced in COVID-19 patients that develop severe disease as compared to mild disease. The quick normalization of glycosylation of anti-spike IgG after seroconversion hints toward aberrant activation of the B cells that are responsible for the first wave of anti-SARS-CoV-2-antibodies, mostly likely the short-lived plasmablasts. Indeed, critically ill COVID-19 patients are characterized by extrafollicular B cell activation, which coincides with early production and high concentrations of SARS-CoV-2-specific neutralizing antibodies (*54*). The molecular processes that underlie the production of IgG with aberrant glycosylation in these cells is still unclear, but could be related to increased endoplasmic reticulum stress or different expression of proteins such as Jagunal homolog 1 (*55*). For future studies, it would be very interesting to study how risk factors of severe COVID-19 (such as age, obesity, and co-morbidities) impact these glycosylation processes in B cells. In addition to IgG, the extrafollicular B cells also produce IgM and IgA (*54*). Whether these isotypes are also aberrantly glycosylated in severe COVID-19 patients is still unknown. However, particularly antibodies of the IgA isotype can promote inflammation depending on the glycosylation profile (*56*). On top of this, IgG subclasses (IgG1-4) could also play a role in both the amplitude and the kinetics of anti-spike-induced inflammatory responses (*57*). For example, IgG3 is typically the first IgG subclass to be produced in response to viruses and generally shows a glycosylation pattern similar to IgG1 (*26*, *58*). Finally, also cell-intrinsic differences in macrophages may contribute to increased or decreased susceptibility of particular individuals to IgG glycosylation differences. This could be related to genetic polymorphisms such as *FCGR2A* and *FCGR3A* SNPs or downstream signaling molecules, but could also be related to epigenetic differences in macrophages or their precursors.

A limitation of our current study is that we have not been able to perfectly match mild and severe COVID-19 patients in terms of age and several other parameters because of practical limitations. This study required serum from mild patients quickly after seroconversion, which is generally difficult to obtain since mildly ill COVID-19 patients are not hospitalized (or even diagnosed), and therefore difficult to follow over time. Although here we were able to match for the most important parameter (day of onset), it will be relevant to additionally match for age, sex, body mass index, and co-morbidities in future studies. In addition, it is not yet clear if ADI is specific for severe SARS-CoV-2 infection, or whether it may also occur upon infection with other viruses. Although induction of afucosylated IgG may be a common trait of enveloped viruses (*28*), excessive inflammation right after seroconversion appears to be rare event for most viral infections in humans, with the exception of SARS-CoV-1 (*14*). Yet, theoretically, ADI may still occur during other viral infections, but in a less pronounced manner that does not lead to pathology. Finally, it is important to realize that, although we specifically focused on ADI in this study, antibodies have additional effector functions that will be activated simultaneously in COVID-19 patients. Whereas over-activation of ADI leads to pathology, increased activation of ADCC or phagocytosis of infected cells by afucosylated IgG could simultaneously have beneficial effects such as increasing viral clearance. Therefore, in future work it will be interesting to determine how afucosylation affects other anti-viral IgG effector functions in COVID-19 patients.

We here showed that the observed inflammatory response induced by anti-spike IgG from severe patients could be specifically counteracted by the Syk inhibitor R406, the pro-drug of fostamatinib. Notably, fostamatinib is an FDA- and EMA-approved drug that is currently used for treatment of ITP (*37*), which may facilitate repurposing for the treatment of severe COVID-19 patients. A recent study indicates that fostamatinib may also counteract acute lung injury by inhibiting Mucin-1 expression on epithelial cells, suggesting that fostamatinib may target multiple pathways simultaneously (*59*). In addition to fostamatinib, also other drugs that interfere with FcγR activation could be efficacious to counteract anti-spike IgG-induced inflammation in COVID-19 patients. Previous studies already showed beneficial effects of treatment with intravenous immunoglobulin (IVIG), which can interfere with FcγR activation (*60*). Alternatively, it could be interesting to target critical molecules in FcγR downstream signaling. For example, the Syk-dependent FcγR signaling pathway critically depends on the transcription factor interferon regulatory factor (IRF) 5 (*17*, *36*), which can be targeted using cell-penetrating peptides (*61*). Furthermore, FcγR stimulation is known to induce metabolic reprogramming of human macrophages (*36*), which is also observed in patients with COVID-19 (*62*), and therefore may provide additional targets for therapy. These findings may not only be valuable to find new ways to treat the most severely ill COVID-19 patients, but may also have implications for the therapeutic use of convalescent serum, for which it may be wise to omit the afucosylated IgGs that are present in severely ill patients. Similarly, for recombinant neutralizing antibodies the composition of the Fc tail needs to be carefully considered, since extreme activation of Fc effector functions by afucosylation needs to be prevented, while at the same time Fc effector functions should remain partially intact to provide optimal therapeutic protection (*63*). Since ADI appears to lead to excessive inflammation upon infection with both SARS-CoV-1 (*14*) and SARS-CoV-2 viruses, these findings may also be relevant in case of emergence of a future outbreak with related coronaviruses. In conclusion, our data indicate a pathogenic role for anti-SARS-CoV-2 antibodies in patients who are severely ill with COVID-19 caused by high titers and low fucosylation of anti-spike IgG. Moreover, we define therapeutically relevant approaches to suppress the induced cytokine release. These data thus warrants future investigations into the therapeutic potential of targeting this inflammatory mechanism in patients with COVID-19.

## MATERIALS AND METHODS

### Study design

The study was designed to investigate the effect of SARS-Cov-2 immune complexes on macrophage activation and clinically relevant in vitro parameters. We applied a human monocyte-derived macrophage model of IL-10 polarized macrophages, resembling human alveolar macrophages (*16*). We verified these data in primary human macrophages obtained via BAL. In the studies we analyzed sera from patients hospitalized at Amsterdam UMC Intensive Care Units (n=27) and compared these to sera from ICU patients negative for SARS-Cov-2 (n=2), sera from patients positive for SARS-Cov-2 but negative for IgG against spike protein (n=4) and to the response induced by recombinant anti-spike IgG, COVA1-18 (*24*). The COVID-19 patients were included based on serology (positive for anti-spike), except for the control COVID-19 patients in Fig. 1D, which needed to have a positive quantitative (q)PCR result but also had to be seronegative. No other selection criteria were used and there were no outliers. For the comparison with mild patients we worked with sera from patients that tested positive for SARS-Cov-2 but were not hospitalized (n=10). Mild patients sera were selected by matching gender and serum collection date as comparable as possible with the ICU sera. Smaller subsets of sera were used for selected experiments as described in the respective methods. Cytokine production assays were repeated at least in two donors. Investigators were not blinded for the patient status of the serum used. Samples were randomly assigned to positions in culture plates.

### Cells

Buffy coats from healthy anonymous donors were acquired from the Sanquin blood supply in Amsterdam, the Netherlands. All the subjects provided written informed consent prior to donation to Sanquin. Monocytes were isolated from the Buffy coats through density centrifugation using Lymphoprep (Axis-Shield) followed by human CD14 magnetic beads purification with the MACS cell separation columns (Miltenyi Biotec) as previously described (*16*). The resulting monocytes were seeded on tissue culture plates and subsequently differentiated to macrophages for 6 days in the presence of 50ng/mL human M-CSF (Miltenyi Biotec) with Iscove's Modified Dulbecco's Medium (IMDM, Lonza) containing 5% fetal bovine serum (FBS, Biowest) and 86μg/mL gentamicin (Gibco). The medium was renewed on the third day. After a 6 day differentiation period, the medium was replaced by culture medium without M-CSF and supplemented with 50 ng/mL IL-10 (R&D Systems) for 24 hours to generate alveolar macrophage-like monocyte-derived macrophages. These macrophages were then detached with TrypLE Select (Gibco) for further treatment and stimulation.

Pulmonary artery endothelial cells (PAEC) were obtained from resected pulmonary artery tissue, obtained from lobectomy surgery performed at Amsterdam UMC and isolated according to the previously published protocol (*35*). Briefly, the endothelial cell layer was carefully scraped onto fibronectin-coated (5 μg/mL) culture dishes (Corning, #3295), and maintained in culture in endothelial cell medium (ECM, ScienCell, #1001) supplemented with 1% Penicillin/Streptomycin, 1% endothelial cell growth supplement (ECGS), 5% FBS, and 1% non-essential amino acids (NEAA, Biowest, #X055-100). Cells were grown until passage 4-6 for experiments.

Primary macrophages were prepared from BAL fluid that was obtained as spare material from the ongoing DIVA study (Netherlands Trial Register: NL6318; AMC Medical Ethical Committee approval number: 2014_294). The DIVA study includes healthy male volunteers aged 18-35. In this study, the subjects are given lipopolysaccharide (LPS) intravenously and, two hours later, a dose of either fresh or aged platelet concentrate or NaCl 0.9% intravenously. Six hours after the platelet or NaCl treatment, a BAL was performed by a trained pulmonologist according to national guidelines. Fractions 2-8 were pooled and split in two, one half is centrifuged (4°C, 1750 × g, 10 min), the cell pellet of which was used in this research. Since the COVID-19 pandemic, subjects are also screened for SARS-CoV-2 via throat swab polymerase chain reaction (PCR) 2 days prior to the BAL. All subjects in the DIVA study have signed an informed consent form. The frequency of macrophages (80-85%) in the BAL was determined by counting the cells that did not adhere to the plate after 30 min at 37°C. For our experiments, complete cell pellets were stimulated.

### Coating

To mimic spike protein specific immune complexes, 2μg/mL soluble prefusion-stabilized spike proteins of SARS-CoV-2 was coated overnight on a 96-well high-affinity plate (Nunc). Plate were blocked with 10% fetal calf serum (FCS) in phosphate buffered saline (PBS) for 1 hour at 37°C. Then diluted serum or 2 μg/mL anti-SARS-CoV-2 monoclonal antibodies or purified IgG were added and incubated for 1 hour at 37°C. The spike and anti-SARS-CoV-2 monoclonal antibody COVA1-18 were generated as described previously (*24*). When using anti-D glyco-variants, 2 μg/mL soluble antibodies were coated overnight on a 96-well high-affinity plate (Nunc) and plates were blocked afterwards with 10% FCS in PBS for 1 hour at 37°C. Patient sera were provided by the Amsterdam UMC COVID-19 Biobank based on a deferred consent procedure for the usage of materials and clinical information for research purposes, approved by the medical ethics committees of Amsterdam University Medical Centers. COVID-19 patients were included based on serology (positive for anti-spike), except for the control COVID-19 patients in Fig. 1D, which needed to have a positive quantitative (q)PCR result but also had to be seronegative. Severe COVID-19 patients were defined as hospitalized at the ICU, whereas mild patients were defined as symptomatic, but not hospitalized.

The anti-D glyco-variant antibodies were made as previously described (*64*).The specific glyco-engineered antibodies were made from the potent SARS-CoV-2 neutralizing antibody COVA1-18 produced in 293F cells as previously described (*24*). Glyco-engineering tools were used to alter N-linked glycosylation of the N297 glycan in the Fc domain and thereby generated several COVA1-18 glycoforms (*65*). To decrease fucosylation of the N-linked glycan 0.2mM of the decoy substrate for fucosylation, 2-deoxy-2-fluoro-l-fucose (2FF) (Carbosynth, MD06089) was added one hour prior to transfection. To produce a COVA1-18 variant with elevated galactosylation, 293F cells were co-transfected (1% of total DNA) with a plasmid expressing Beta-1,4-Galactosyltransferase 1 (B4GALT1). In addition 5mM D-Galactose was added 1 hour before transfection. Antibodies were purified with protein G affinity chromatography as previously described (*24*) and stored in PBS at 4°C. To determine the glycosylation of COVA1-18, aliquots of the mAb samples (5μg) were subjected to acid denaturation (100 mM formic acid, 5 min), followed by vacuum centrifugation. Subsequently, samples were trypsinized, and Fc glycopeptides were measured as described previously (*28*). Relative abundances of Fc glycopeptides were determined, and amounts of bisection, fucosylation, galactosylation and sialylation were determined as described before (*28*).

Total IgG from individual donors was purified from about 10 μL of serum diluted in PBS using the AssayMAP Bravo platform (Agilent Technologies) with Protein G-coupled cartridges as described elsewhere (*28*). Samples were eluted into neutralization buffer (Tris (214 mM); Na_2_HPO_4_ (22 mM)) to obtain neutral pH. Concentrations of purified IgG was determined by absorbance at 280 nm (NanoDrop, Thermo Fisher Scientific)

### Titer determination

Total IgG to receptor binding domain (RBD) was measured as described previously (*66*), using RBD proteins as described in Vogelzang *et al*. (*67*)*.* In short, MaxiSORP plates were coated with 1.0 μg/mL RBD in PBS overnight. After washing samples were diluted 10,800-fold in PBS supplemented with 0.02% polysorbate-20 and 0.3% gelatin (PTG) and incubated for 1 hour at room temperature. After washing 0.5 μg/mL horseradish peroxidase (HRP)-conjugated monoclonal mouse-anti-human IgG (MH16, Sanquin) was added for 1 hour at room temperature, diluted in PTG. Afterwards, enzymatic conversion of the tetramethylbenzidine (TMB) substrate was used to evaluate antibody binding by measuring the difference in absorbance at 450 nm and 540 nm. Antibody binding was quantified using a serially diluted calibrator consisting of pooled convalescent plasma that was included on each plate. This calibrator was arbitrarily assigned a value of 100 AU/mL. Results were expressed as arbitrary units per mL (AU/mL) and represent a semiquantitative measure of the concentrations of IgG antibodies.

### Stimulation

Macrophages (50,000/well) were stimulated in a pre-coated plates as described above in combination with 20 μg/mL poly(I:C) (Sigma-Aldrich), 100 ng/mL LPS (from *Escherichia coli* o111:B4, Sigma-Aldrich), 5 μg/mL CL097 (InvivoGen), 100 ng/ml R848 (Sigma-Aldrich), or 10 μg/mL Pam3CSK (InvivoGen). To block Syk, cells were pre-incubated with 0.5 μM R406 (Selleckchem) or dimethyl sulfoxide (DMSO) (Sigma-Aldrich) as a control, for 30 min at 37°C. To block the different FcRs, cells were pre-incubated with 20 μg/mL of the following antibodies: (anti-FcyRI (CD64; 10.1; BD Biosciences); anti-FcyRIIa (CD32a; IV.3; StemCell Technologies); anti-FcyRIII (CD16; 3G8; BD Biosciences); and anti-FcαRI (CD89; MIP8a; Abcam)) for 30 min at 4°C. Then media was added to a final antibody concentration of 5 μg/mL.

### Virus and HEK293F opsonization

To mimic opsonized SARS-CoV-2 or infected cells, SARS-CoV-2 pseudovirus or SARS-CoV-2 spike-expressing HEK293F cells were generated. Transient transfection of HEK293F cells with SARS-2 spike was performed as previously described (*24*). To obtain spike surface expression, 62.5 mL HEK293F cells (at a density of 1E6/mL) were transfected with 20 μg of SARS-CoV-2 full-length spike plasmid DNA and 60 μg PEImax. After 60-72 hours, cells were harvested, and pre-incubated with COVA1-18. Then HEK297F cells were three times washed and added to the macrophages in a 1:1 ratio in combination with or without poly(I:C). After 24 hours, supernatant was harvested and cytokine production was assessed with enzyme-linked immune sorbent assay (ELISA). To produce a SARS-CoV-2 S-pseudo-typed HIV-1 virus, a SARS-CoV-2 spike expression plasmid was co-transfected in HEK293T cells (ATCC, CRL-11268) with an HIV backbone expressing firefly luciferase (pNL4-3.Luc.R-E-) as previously described (*24*). After three days culture supernatants were harvested and stored at -80°C. To quantify pseudovirus production and determine the viral input for the macrophage activation assay, a capsid p24 antigen ELISA was performed (*68*). Monocyte-derived macrophages were incubated in 96-wells flat-bottom plates at 37°C with SARS-CoV-2 pseudo-typed particles (an equivalent of 0.2 ng CA p24 antigen) in the presence or absence of 20 μg/mL poly(I:C) (Sigma-Aldrich) and 0.4 ng/mL COVA1-18 antibody. After 24 hours, supernatant was harvested and cytokine production was assessed by ELISA.

### Endothelial barrier function

PAEC passage 4-6 cells were seeded 1:1 in 0.1% gelatin coated 8-well (8W10E) or 96-well (96W10idf PET) IBIDI culture slides for electrical cell-substrate impedance sensing (ECIS), as previously described (*34*). Cells were maintained in culture in endothelial cell medium (ECM, ScienCell, #1001) supplemented with 1% Penicillin/Streptomycin, 1% endothelial cell growth supplement (ECGS), 5% FBS and 1% non-essential amino acids (NEAA, Biowest, #X055-100), with medium change every other day. From seeding onwards, electrical impedance was measured at 4000Hz every 5 min. Cells were grown to confluence and after 72 hours, ECM medium was removed and replaced by supernatant of alveolar macrophage-like monocyte-derived macrophages stimulated for 6 hours as described above with poly(I:C), or in combination with patient serum. Within every experiment triplicate measurements were performed for each condition. For every experiment PAECs and macrophages obtained from different donors were used.

### Platelet adhesion on PAEC under flow

PAECs were seeded in 0.1% gelatin coated μ-Slide VI 0.4 ibiTreat flow slides (ibidi, #80606) and cultured for 7 days. PAECs were pre-incubated for 24 hours with supernatant of alveolar macrophage-like monocyte-derived macrophages stimulated for 6 hours as described above with poly(I:C), or in combination with patients serum before flow experiments were performed. On the day of perfusion, citrated blood was collected from healthy volunteers and platelets were isolated as previously described (*69*). Platelets were perfused for 5 min and phase-contrast and fluorescent images were taken with an Etaluma LS720 microscope using a 20X phase-contrast objective. Platelet adhesion was quantified in ImageJ by determining the area covered by platelets per Field of View (FOV).

### ELISA

To determine cytokine production, supernatants were harvested after 24 hours of stimulation and cytokines were detected using the following antibody pairs: IL-1β and IL-6 (U-CyTech Biosciences); TNF (eBioscience); and IL-8 (Invitrogen). Concentration of (anti-spike) antibodies present in patients serum was determined as described before (*28*).

Flow supernatant was collected after perfusion and von Willebrand Factor concentrations were measured with ELISA. An 96-well high affinity ELISA plate was coated with polyclonal anti-von Willebrand Factor (1:1000, Dako, #A0082) and blocked with 2% bovine serum albumin. Samples were loaded and bound von Willebrand Factor was detected with HRP-conjugated rabbit polyclonal anti- von Willebrand Factor (1:2500, Dako, #A0082). Normal plasma with a stock concentration of 50 nM von Willebrand Factor (gifted from Sanquin) was used as a standard for determination of concentration, measured at 450nm and 540nm.

### qPCR

Total RNA was isolated with RNeasy Mini Kit (Qiagen) and RNase-Free DNase Set (Qiagen) per the manufacturer's protocol. RNA was then converted to cDNA with iScript (Life Technologies). qPCR was performed with Sybr Green Fast on a ViiA7 PCR machine (Applied Biosystems). All genes were normalized to the mean of the relative expression values of two housekeeping genes, *HPRT1 and RACK1.*

*IL6*: (hsIL6-FW GAGTAGTGAGGAACAAGCCAG, hsIL6-RV TTGTCATGTCCTGCAGCC)

*IL8 (CXCL8)*: (hsIL8-FW ATACTCCAAACCTTTCCACC, hsIL8-RV TCCAGACAGAGCTCTCTTCC)

*TNF*: (hsTNFa-FW GGCGTGGAGCTGAGAGAT, hsTNFa-RV TGGTAGGAGACGGCGATG)

*CXCL10*: (hsCXCL10-FW GAAAGCAGTTAGCAAGGAAAGGT, hsCXCL10-RV GACATATACTCCATGTAGGGAAGTGA)

*HPRT1*: (hsHPRT1_FW GACCAGTCAACAGGGGACAT, hsHPRT1_RV AACACTTCGTGGGGTCCTTTTC)

*RACK1*: (hsRACK1_FW GAGTGTGGCCTTCTCCTCTG, hsRACK1_RV GCTTGCAGTTAGCCAGGTTC)

*CD163:* (hsCD163_FW AGTCCCAAACACTGTCCTCG, hsCD163_RV GGCGAAGTTGACCACTCTCTAT)

*CD209*: (hsCD209_FR AATGGCTGGAACGACGACAAA, hsCD209_RV CAGGAGGCTGCGGACTTTTT)

### Meso Scale Discovery multiplex assay

V-PLEX Custom Human Cytokine10-plex kits for Proinflammatory Panel1 and Chemokine Panel 1 (K151A0H-2, for IL-1β, IL-6, IL-8, IL-10, TNF, CCL2, CXCL10) and U-PLEX human Interferon Combo SECTOR (K15094K-2, for IFN-α2a, IFN-β, IFN-γ, IFN-λ1) were purchased from Mesoscale Discovery (MSD). The lyophilized cocktail mix calibrators for Proinflammatory Panel 1, Chemokine Panel 1, and 4 calibrators for U-PLEX Biomarker Group 1 (Calibrator 1, 3, 6, 9) were reconstituted in provided assay diluents respectively. U-PLEX plates were coating with supplied linkers and biotinylated capture antibodies according to manufacturer’s instructions. Proinflammatory cytokines and chemokines in supernatant collected at 24 hours after stimulation were detected with pre-coated V-PLEX and interferons in 6 hour supernatant were measured by coated U-PLEX plates. The assays were performed according to manufacturer’s protocol with overnight incubation of the diluted samples and standards at 4°C. The electrochemiluminescence signal (ECL) were detected by MESO QuickPlex SQ 120 plate reader (MSD) and analyzed with Discovery Workbench Software (v4.0, MSD). The concentration of each sample was calculated based on the four-parameter logistic fitting model generated with the standards (concentration was determined according to the certificate of analysis provided by MSD). log_10_ values of measured concentrations of IL-1β, IL-6, IL-8, IL-10, TNF, IFN-β, IFN-γ, CXCL10 were used for the principle component analysis. log_10_ IgG titers (half maximal effective concentration, EC_50_) were used for the color overlay.

### RNA Sequencing

Cells were stimulated as described above and lysed after 6 hours. Total RNA was isolated with RNeasy Mini Kit (Qiagen) and RNase-Free DNase Set (Qiagen) per the manufacturer's protocol. cDNA libraries were prepared using the standard protocol of KAPA mRNA HyperPrep Kits (Roche) with input of 300ng RNA per sample. Size-selected cDNA libraries were pooled and sequenced on a HiSeq 4000 sequencer (Illumina) to a depth of 16-20M per sample according to the 50 bp single-end protocol at the Amsterdam University Medical Centers, location Vrije Universiteit medical center. Raw FASTQ files were aligned to the human genome GRCh38 by STAR (v2.5.2b) with default settings (*70*). Indexed Binary alignment map (BAM) files were generated and filtered on MAPQ>15 with SAMTools (v1.3.1) (*71*). Raw tag counts and reads per kilo base million (RPKM) per gene were calculated using HOMER2’s analyzeRepeats.pl script with default settings and the -noadj or -rpkm options for raw counts and RPKM reporting (*72*) for further analyses.

### Flow cytometry

After detachment, macrophages were stained with antibodies against Fc gamma receptors: FcγRI (CD64; cat# 305014, BioLegend), FcγRII(CD32; cat# 555448, BD Biosciences), and FcγRIII (CD16; cat# 562293, BD Biosciences). Fluorescence was measured with CytoFLEX Flow Cytometer and analyzed with FlowJo software version 7.6.5 (FlowJo, LLC). Fluorescence Minus One (FMO) controls were used for each staining as negative controls.

### Functional analyses of transcriptomic data

All analyses were performed in the R statistical environment (v3.6.3). Differential expression was assessed using the Bioconductor package edgeR (v3.28.1) (*73*). Lowly expressed genes were filtered with the filterByExpr function and gene expression called differential with a false discovery rate (FDR) <0.05. Pathway enrichment analyses were performed on the differentially regulated genes with an absolute log_2_(fold change) higher than 1 using the Metascape (http://metascape.org/gp/index.html) (*74*) on 2020-06-26. For heatmaps, normalized expression values (count per million, CPM) of each gene were calculated and plotted using pheatmap (v1.0.12) with values scaled by gene. Gene set enrichment analysis (GSEA) was performed with Bioconductor package fgsea (v1.12.0) (*75*) with genes ranked by effect size (Cohen’s *d*) with respect to the “R406+serum+spike+poly(I:C) vs serum+spike+poly(I:C)” against the curated gene sets obtained from gene ontology (GO) by Bioconductor package biomaRt (v2.42.1) (*76*). A total of 5000 permutations were performed to estimate the empirical *P* values for the gene sets. Normalized enrichment scores and the Benjamini-Hochberg (BH)-adjusted *P* values were provided in the figure. De novo transcription factor motif analysis was performed by using HOMER (v4.11) (*77*) using the following parameters: -start -200 -end 100 -len 8, 10, 12.

### Statistical analysis

Statistical significance of the data was performed in GraphPad Prism version 8 (GraphPad Software). For *t* tests or nonparametric analysis comparing two sets of measurements, data were first examined with D'Agostino-Pearson normality test with an alpha = 0.05. For the data following normal distribution, paired or unpaired *t* tests were conducted based on the experiment design. For unpaired data not following a normal distribution, Mann-Whitney test was applied. For multiple comparison tests, one-way or two-way ANOVA was applied based on the addressed scientific question. Brown-Forsythe and Welch’s ANOVA test was applied when not assuming that the compared groups were sampled from populations with equal variances (examined by Brown-Forsythe test), otherwise an ordinary one-way ANOVA was performed. For differential analysis and gene set enrichment analysis of transcriptomic data, P values were adjusted by BH procedure to control the FDR. The analysis methods applied for each figure were stated in the legends.

## Supplementary Materials

stm.sciencemag.org/cgi/content/full/scitranslmed.abf8654/DC1 Fig. S1 to S4 Table S1 to S3 Data File S1. Raw Data.
